# Assessment of the Level of Knowledge and Awareness of the Risk of Chronic Use of Steroids in Causing Cataracts Among the General Population in the Western Region of the Kingdom of Saudi Arabia (KSA): A Cross-Sectional Study

**DOI:** 10.7759/cureus.52288

**Published:** 2024-01-15

**Authors:** Ashjan Bamahfouz, Soha Elmorsy, Elham R Alharbi, Salah M Bakry, Waleed Alnemari, Sulten M Alzahrani, Amr A Almousa

**Affiliations:** 1 Ophthalmology, Umm Al-Qura University, Makkah, SAU; 2 Ophthalmology, King Abdullah Medical City, Makkah, SAU; 3 Ophthalmology, Security Forces Hospital-Makkah, Makkah, SAU; 4 Research and Development, King Abdullah Medical City, Makkah, SAU; 5 Ophthalmology, Al-Noor Specialist Hospital, Makkah, SAU; 6 Faculty of Medicine, Umm Al-Qura University, Makkah, SAU; 7 Medicine and Surgery, Umm Al-Qura University, Makkah, SAU

**Keywords:** saudi arabia, western region, steroid therapy, cataract, awareness, knowledge

## Abstract

Background/Aim: Cataracts consequence blindness to burden and impose health and economic burdens on communities. Steroid-induced cataracts have scarcely been highlighted in previous literature, creating a demand for reinvestigating this issue among the general population of western Saudi Arabia.

Methods: A cross-sectional study was conducted in 2022 using an online survey distributed among the target participants via social media platforms. The data were gathered and statistically analyzed using IBM Corp. Released 2015. IBM SPSS Statistics for Windows, Version 23.0. Armonk, NY: IBM Corp. software.

Results: Overall, 866 respondents (males = 42.5%, females = 57.5%) were enrolled in this survey (mean age = 28.08, SD = 13). The participants' correct responses to questions about steroid-induced cataracts showed inadequate representation (below 50%). Additionally, most of the participants (94.23%) had a poor understanding of steroid-induced cataracts.

Conclusion: The participants' level of understanding of the risk factors associated with chronic steroid usage and its impact on cataracts was inadequate.

## Introduction

Good vision is an essential aspect of an autonomous life. Globally, at least one billion people have near or distant vision impairments that have been prevented or have not yet been treated [1,2]. Uncorrected refractive errors are the primary causes of visual impairments, followed by cataracts [1].

A cataract is an opacity of the transparent eye lens, and this condition can be physiological (due to aging) or pathological [3]. It is mainly treated by steroids, a group of anti-inflammatory drugs commonly used to treat ocular and systemic conditions [4,5]. Steroid therapy is the fourth main risk factor for secondary cataracts, representing approximately 4.7% of all cataracts worldwide [6]. Using steroids for more than four months per year can lead to significant ocular side effects, including posterior subcapsular cataracts (PSCs) and increased intraocular pressure (IOP) [4,7]. Chronic administration of steroids in any form with raised IOP can cause optic neuropathy, resulting in steroid-induced glaucoma [4]. IOP elevation in patients using steroids usually takes a few weeks to develop. However, it can rise within hours and usually normalizes within 1 to 4 weeks after the steroid is discontinued [4].

In the general population of the Kingdom of Saudi Arabia (KSA), there is a lack of knowledge about the impacts of long-term steroid therapy on cataracts. This points to the urgent need for future studies to increase awareness and prevent the occurrence of cataracts in the KSA [6,8]. Therefore, this survey aims to highlight and estimate the level of knowledge of cataracts induced by steroid use in western Saudi Arabia.

## Materials and methods

Study design and participants

An online survey-based cross-sectional study was conducted from October 2021 to May 2022. Male and female residents of the KSA's western region (Makkah, Jeddah, Taif, and Al-Qunfudah) between the ages of 18 and 65 were invited to participate in this study. Those under 18 or over 65 years old and from outside the western region were disqualified. The people who declined to participate in this study were also deleted from the list of eligible respondents. By utilizing non-probability sampling methods, we estimated the sample size using the Epi Info software, version 2.1 [9]. To obtain a 95% confidence interval and a 5% acceptable error margin, the minimum sample that we had to collect was 384. To cover for incomplete participation, we included 866 during the data collection.

Survey design and study procedure

Twenty closed-ended items were included in the two portions of the self-administered survey that were translated into Arabic. Three questions made up the first portion, which was mostly concerned with participant social-demographic data. The following sections, which had 12 questions with a combination of alternatives of the types yes, no, and don't know, were highlighted in the comprehension of steroid-induced cataracts.

Before the survey, 10 respondents were sent questionnaire drafts, and their feedback was gathered to reduce specialized medical terminology and reconstruct some survey topics. However, the results of the pilot research were not considered in the final statistical analysis. To ensure that errors did not affect the research tool, a reverse translation from Arabic to English was also done.

The participants were informed about this study through social media sites such as Facebook, Twitter, Instagram, Snapchat, and WhatsApp. In light of the most recent research, this poll was modified from earlier studies [6,8]. The corresponding author's email was written to answer any matter, and we obtained online informed consent from all participants.

Ethical considerations

The Umm Al-Qura ethical committee granted its ethical approval for this survey in 2022, which was conducted under the Declaration of Helsinki's principles. The participants' names, phone numbers, and identity card numbers were not included to maintain anonymity and confidentiality. All respondents provided their online informed consent before the survey and were made aware that the survey was voluntary, confidential, and intended for academic purposes only. 

Statistical analysis and scoring system

The obtained data were initially entered in an Excel sheet to be checked. Afterward, we used the IBM Corp. Released 2015. IBM SPSS Statistics for Windows, Version 23.0. Armonk, NY: IBM Corp. for the data analysis, calculating the mean standard deviation (SD) and significance utilizing the chi-square test, with a p-value of < 0.05 to be considered statistically significant. 

A modified Bloom's criteria scoring system cut-off value of 75% [9] was used to analyze the data, which was coded and inputted into the SPSS software. For questions estimating the level of understanding, a score of one was given for "yes," while a score of zero was given for "no" and "don't know."

## Results

We conducted an online, self-administered survey of the participants from the Makkah region. Eight hundred sixty-six participants were enrolled in this survey, with a mean age of 28.08 (SD = 13) and 21-30-year-old participants comprising the largest age group (n = 387, 44.7%). Females comprised the majority of the respondents (n = 498, 57.5%). Participants with a university degree were predominantly represented (n = 603, 69.6%) (Table 1).

**Table 1:** Participants’ demographic profiles .

Variables	Categories	N	%
Age groups	12-20	244	28.2%
	21-30	387	44.7%
	31-40	93	10.7%
	41-50	56	6.5%
	51-60	60	6.9%
	61-70	25	2.9%
	71-90	1	0.1%
Gender	Male	368	42.5%
	Female	498	57.5%
Educational level	Primary school	26	3.0%
	Secondary school	237	27.4%
	University	603	69.6%
Ever heard about steroids induced cataract	Yes	535	61.8%
	No	331	38.2%
Previous diagnosed with cataract	Yes	20	2.3%
	No	846	97.7%
Continuous four months use of steroids	Yes	58	6.7%
	No	808	93.3%
Route of steroids uses in previous four months	Topical	39	4.5%
	Oral	21	2.4%
	Intravascular	5	0.6%
	Inhaler	20	2.3%
	Don’t use	781	90.2%
Age (Mean) (standard deviation)	(Mean=28.08) (SD=13.0)		

Most participants had heard about steroid-induced cataracts (n = 535, 61.8%). Only 2.3% of the participants had been previously diagnosed with cataracts, while the majority had a negative history (n = 846, 97.7%). Regarding the participants' compliance with the four-month continuous use of steroids, the majority reported non-compliance with their treatment (n = 808, 93.3%) (Table 1).

Most participants had not used steroids for the previous four months (n = 781; 90.2%). Among those who used steroids over the past four months, most did so topically (4.5%), followed by orally and intravascularly (2.4% and 0.6%, respectively) (Table 1).

**Figure 1:**
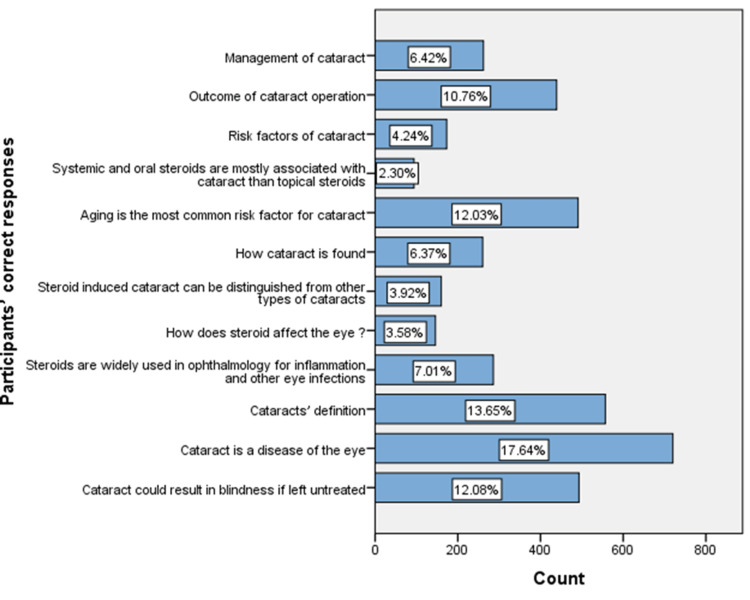
Participants' correct responses of knowledge-related questions

Figure 1 presents the participants' correct answers regarding the risk of cataracts associated with continuous steroid use. Despite the inadequate responses, the cataract definition and its origin showed the highest percentages of correct answers among the participants (17.64% and 13.65%, respectively). At the same time, knowledge of associated medications that increase the risk of cataracts and cataract risk factors had the lowest percentages of correct answers (2.30% and 4.24%, respectively).

**Figure 2:**
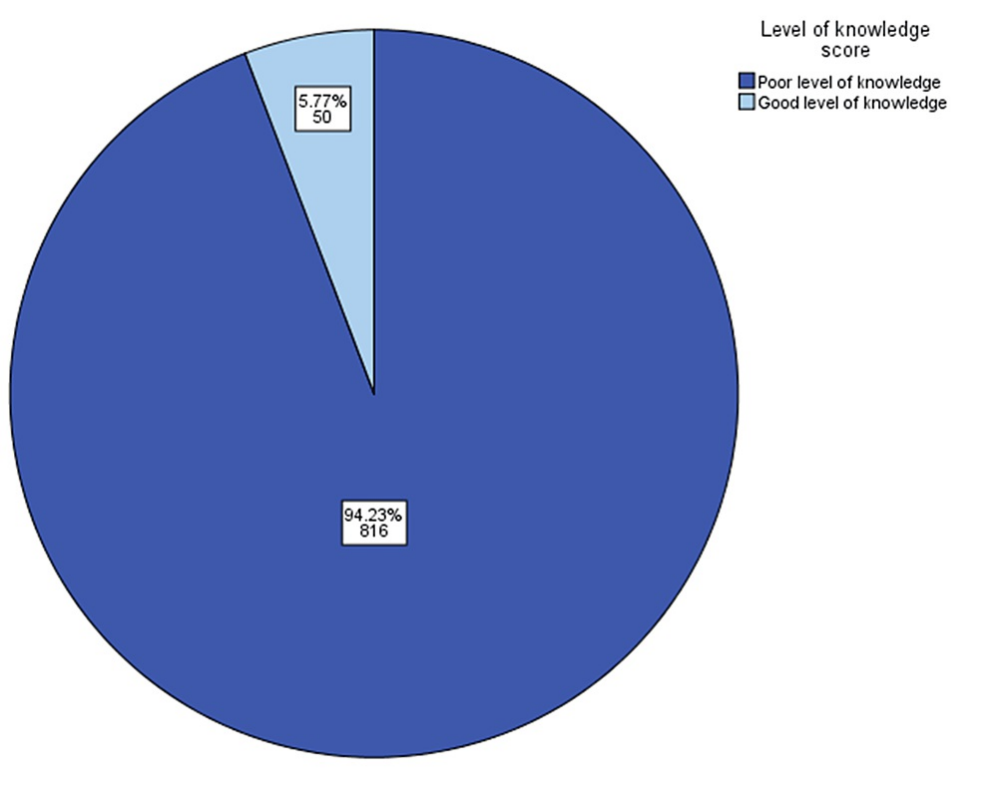
Level of knowledge score

This survey showed that most participants had insufficient knowledge about the risk of cataracts associated with continuous steroid use (n = 816; 94.23%), while the minority had good knowledge (5.77%) (Figure 2).

A significant association was found between the participants who had previous awareness of the term cataract and a poor level of knowledge regarding the risk of cataracts caused by continuous steroid use (n = 43 and 492, respectively) (P-value: < 0.001). However, the participants' age group, gender, and educational level showed an insignificant variation with their level of knowledge (p-values: 0.494, 0.090, and 0.300, respectively). Furthermore, the participants' history of cataracts, history of continuous usage of steroids for up to four months, and route of steroid use in the past four months corresponded to an insignificant association with the level of knowledge (p-values: 0.412, 0.704, and 0.279, respectively) (Table 2).

**Table 2:** The association between participants’ demographic information and their level of knowledge regarding the risk of steroids for cataract

Categories		Level of knowledge		p-value
		Good level of knowledge (N.)	Poor level of knowledge (N.)	
Age groups	12-20	9	235	0.494
	21-30	26	361	
	31-40	5	88	
	41-50	6	50	
	51-60	3	57	
	61-70	1	24	
	71-90	0	1	
Gender	Male	27	341	0.090
	Female	23	475	
Educational level	Primary school	2	24	0.300
	Secondary school	9	228	
	University	39	564	
Ever heard about steroids induced cataract	Yes	43	492	˂0.001*
	No	7	324	
Previous diagnosed with cataract	Yes	2	18	0.412
	No	48	798	
Continuous four months use of steroids	Yes	4	54	0.704
	No	46	762	
Route of steroids uses in previous four months	Topical	1	38	0.279
	Oral (PO)	3	18	
	Intravenous (IV)	0	5	
	Inhaler	0	20	
	Don’t use steroids	46	735	

## Discussion

Due to their links to morbidity, mortality, and lower quality of life, visual impairment and blindness raise serious worldwide health concerns and cause significant economic loss and decreased productivity [1,10]. Understanding common eye conditions and how to avoid and treat them is crucial for encouraging patients to receive timely eye care, which, in turn, helps lessen the burden of vision impairment [1]. Thus, our study aimed to show the level of knowledge regarding the chronic use of steroids and its effect on cataracts in the Makkah region, KSA.

Most of the participants in our study demonstrated inadequate knowledge regarding the steroids' risk of inducing cataracts. This is consistent with Al Khozym's [6] study, which showed most participants' (81.4%) poor level of knowledge. Similarly, in another Saudi study conducted by Aljuaid et al. [8], most of their participants (76.7%) revealed a poor level of knowledge. According to Al Khozym [6], the participants' levels of knowledge in most subjects were inadequate, though they were highly educated. This could be associated with the low prevalence of cataracts in the KSA and the lower prevalence of chronic use of steroids [6].

It has been established that using steroids causes cataracts, glaucoma, and elevated IOP [11]. Additionally, the manner of administration, frequency of application, and length of use of various steroid preparations (oral, topical, depot injections, intravitreal, etc.) might cause eye difficulties [11]. Although steroids are frequently used to treat various disorders, patients must adhere to their doctor's recommendations to prevent the side effects of steroid medication [6,11]. Patients who use steroids for extended periods or in high doses also have a significantly higher risk of developing cataracts [6,12,13].

Few large-scale investigations have been undertaken in the past to evaluate the relationship between inhaled corticosteroids and the development of PSCs and steroid-induced glaucoma [11]. Cumming et al. [7] found that using inhaled cortical steroids (ICS) was linked to the development of PSCs and nuclear cataracts [7,11]. Furthermore, Smeeth et al. [14] discovered that long-term usage of high dosages of ICS was related to an increased risk of cataract formation [11,14].

In contrast to our study's results, Miller et al. [15] found that a fixed-dose combination of fluticasone propionate and salmeterol or other ICS exposure was not linked to an increased risk of cataracts or glaucoma; neither was a dose-response relationship observed in this population-based nested case-control study of COPD patients in the United Kingdom [11,15]. Additionally, Mitchell et al. [16] reported that ICS use was associated with glaucoma in people who had a family history of the condition or increased IOP [11,16].

This study shows some possible limitations. The first is selective or non-responsive bias, which we have overcome by increasing the sample size during the data collection. Another limitation is that this study has no representation among the rest of the Saudi regions. Thus, we recommend further investigation in all Saudi regions.

## Conclusions

The knowledge about the risk factors of chronic usage of steroid therapy and its effects on cataracts was inadequate among most of the participants, although the majority are highly educated. Educational campaigns should be conducted to increase public knowledge about cataracts, which would thus decrease the prevalence of the condition and its associated complications. Awareness about cataracts should be further encouraged among Saudi subjects to improve the outcomes and enhance early screening of the disease and the measures for preventing it.
